# One benefit of COVID‐19 measures in Taiwan: The reduction of influenza infections and severe complications

**DOI:** 10.1111/irv.12778

**Published:** 2020-09-01

**Authors:** Yu‐Lung Hsu, Hsiao‐Chuan Lin, Hsiu‐Mei Wei, Huan‐Cheng Lai, Kao‐Pin Hwang

**Affiliations:** ^1^ Division of Infectious Diseases China Medical University Children’s Hospital China Medical University Taichung Taiwan; ^2^ School of Medicine College of Medicine China Medical University Taichung Taiwan

**Keywords:** COVID‐19, hand hygiene, influenza, mask wearing

1

*Dear Editor,*

Influenza viruses are transmitted through droplets and particle aerosols from the coughs and sneezes of infected people and through contact with surfaces that have been contaminated. In addition to receiving an influenza vaccine, wearing a mask and observing strict hand hygiene are essential to preventing influenza infection.^1^

The annual influenza season in Taiwan is typically from November to March. On January 21, 2020, the fourth week of 2020, Taiwan recorded its first case of COVID‐19. Because the 2003 SARS epidemic was traumatic for Taiwan, most people in Taiwan began practicing strict hand hygiene and wearing masks (regardless of whether they were sick). To prevent an outbreak of COVID‐19, Taiwan's CDC started to amend their mask‐wearing policy, and they encouraged the general public to observe strict hand hygiene and avoid visiting crowded spaces. Although aimed at reducing COVID‐19 transmission, these measures also reduced the transmission of influenza.

We used data on both the 2018 to 2019 and 2019 to 2020 influenza seasons. These data on the weekly number of patients with seasonal influenza were accessed using the open data website of Taiwan's CDC. All patients in Taiwan who have received diagnoses from clinicians—whether on the basis of an observation of clinical symptoms or laboratory findings—have their diagnoses documented in the national health database. Student's *t* test was used to examine the difference in seasonal influenza rates before and after Taiwan's first case of COVID‐19.

**Figure 1:**
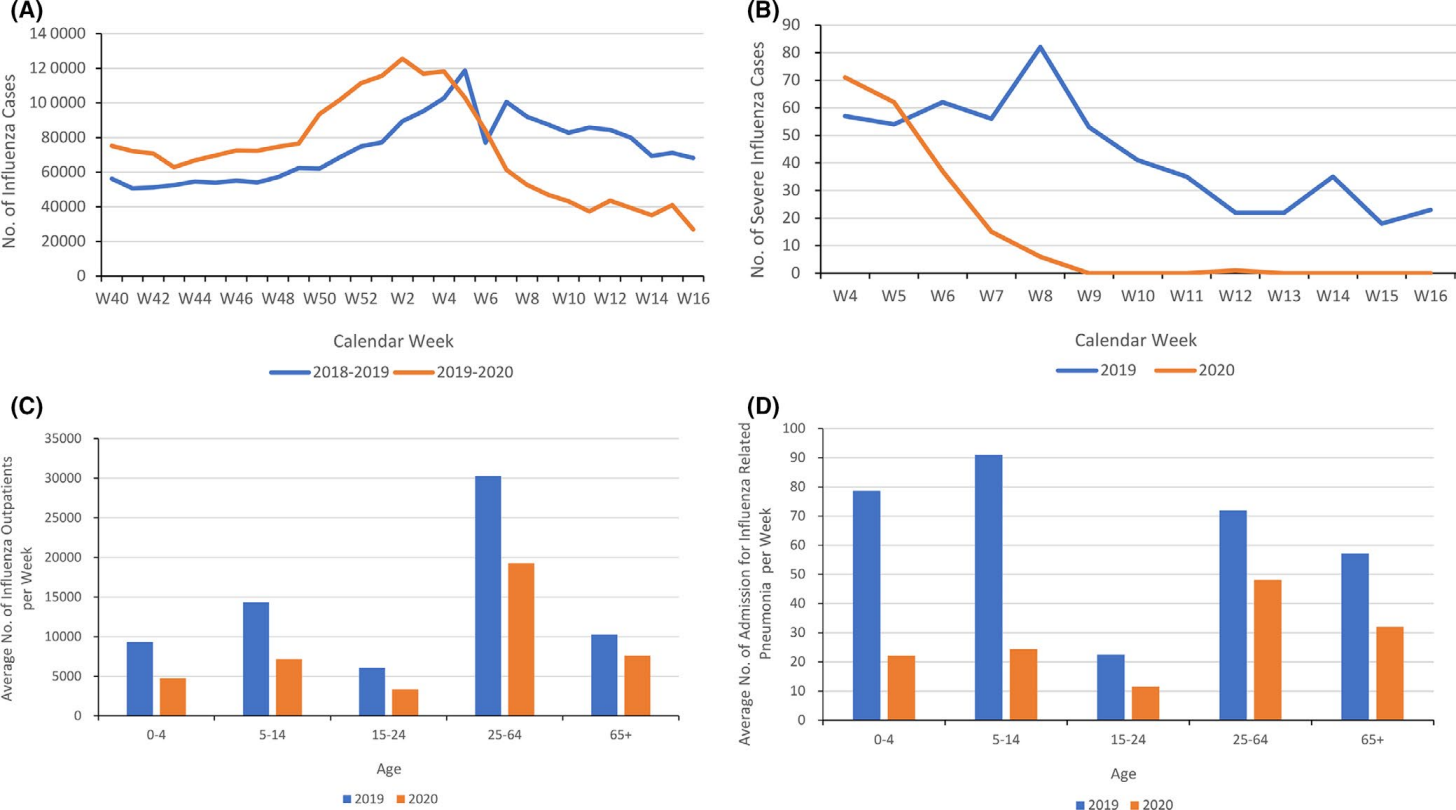
A, Number of patients with Influenza per week in the 2018 to 2019 (blue) and 2019 to 2020 (orange) Influenza seasons. B, Number of patients with severe complications from Influenza per week after the fourth week of 2019 (blue) and 2020 (orange). C, Average number of outpatients with Influenza per Week after the fourth week of 2019 (blue) and 2020 (orange), by Age. D, Average number of patients admitted because of confirmed Influenza‐related Pneumonia per Week After the Fourth Week of 2019 (blue) and 2020 (orange), by age

Relative to the same period in 2019, during the period after Taiwan's first COVID‐19 case, the number of people with influenza per week (86 177 vs 56 379, *P* = .003; Figure 1A) and the number of people with severe complications from influenza (43 vs 15, *P* = .004; Figure 1B) were significantly lower; the number of outpatients with influenza was significantly lower for all age groups (*P* < .01) (Figure 1C), and the number of patients admitted because of confirmed influenza‐related pneumonia per week was also significantly lower especially in younger groups (age < 15 years‐old), except 25 to 64 years old (74 vs 51, *P *= .17; Figure 1D). Relative to the period before the first COVID‐19 case, the number of people with influenza per week (86 167 vs 51 227, *P* < .001; Figure 1A) significantly decreased in the period after.

Consistent with our results, Sakamoto et al noted lower seasonal influenza activity following the COVID‐19 outbreak in Japan.^2^ Our results were documenting the large decrease for all age groups especially in younger groups not only in weekly outpatients with influenza but also admissions due to confirmed influenza‐related pneumonia.

The influenza vaccine is the most effective means of preventing influenza, and Taiwan's influenza vaccine policy has remained unchanged. The influenza vaccine also closely matched the dominant influenza virus during these 2 influenza seasons. Our results also indicated that strict personal hygiene habits, especially emphasizing in children and adolescents, are key to preventing influenza transmission. Therefore, one benefit of COVID‐19 measures in Taiwan is the reduction of influenza infections and complications.

### AUTHOR CONTRIBUTIONS

**Yu‐Lung Hsu:** Conceptualization (lead); Data curation (equal); Formal analysis (equal); Investigation (equal); Methodology (equal); Project administration (equal); Writing‐original draft (lead); Writing‐review & editing (equal). **Hsiao‐Chuan Lin:** Conceptualization (equal); Formal analysis (equal); Methodology (equal); Validation (equal); Writing‐original draft (equal); Writing‐review & editing (equal). **Hsiu‐Mei Wei:** Conceptualization (equal); Data curation (equal); Formal analysis (equal); Writing‐review & editing (equal). **Huan‐Cheng Lai:** Conceptualization (equal); Data curation (equal); Formal analysis (equal); Writing‐review & editing (equal). **Kao‐Pin Hwang:** Conceptualization (equal); Methodology (equal); Supervision (lead); Validation (lead); Writing‐original draft (equal); Writing‐review & editing (lead).

REFERENCES1

Uyeki
TM
, 
Bernstein
HH
, 
Bradley
JS
, et al. Clinical Practice Guidelines by the Infectious Diseases Society of America: 2018 update on diagnosis, treatment, chemoprophylaxis, and institutional outbreak management of seasonal influenzaa. Clin Infect Dis. 2018;68(6):e1‐e47.10.1093/cid/ciy866PMC6653685305665672

Sakamoto
H
, 
Ishikane
M
, 
Ueda
P
. Seasonal influenza activity during the SARS‐CoV‐2 outbreak in Japan. JAMA. 2020;323(19):1969.3227529310.1001/jama.2020.6173PMC7149351
